# Supplementary material for: Biofilm Production Ability, Virulence and Antimicrobial Resistance Genes in Staphylococcus aureus from Various Veterinary Hospitals
Source: Pathogens. 2020 Apr 4;9(4):264. doi: 10.3390/pathogens9040264 (PMC7238219; doi:10.3390/pathogens9040264)
Supplement: Supplementary file 1 [file pathogens-09-00264-s001.zip › Figure S1.pptx]

## Slide 1
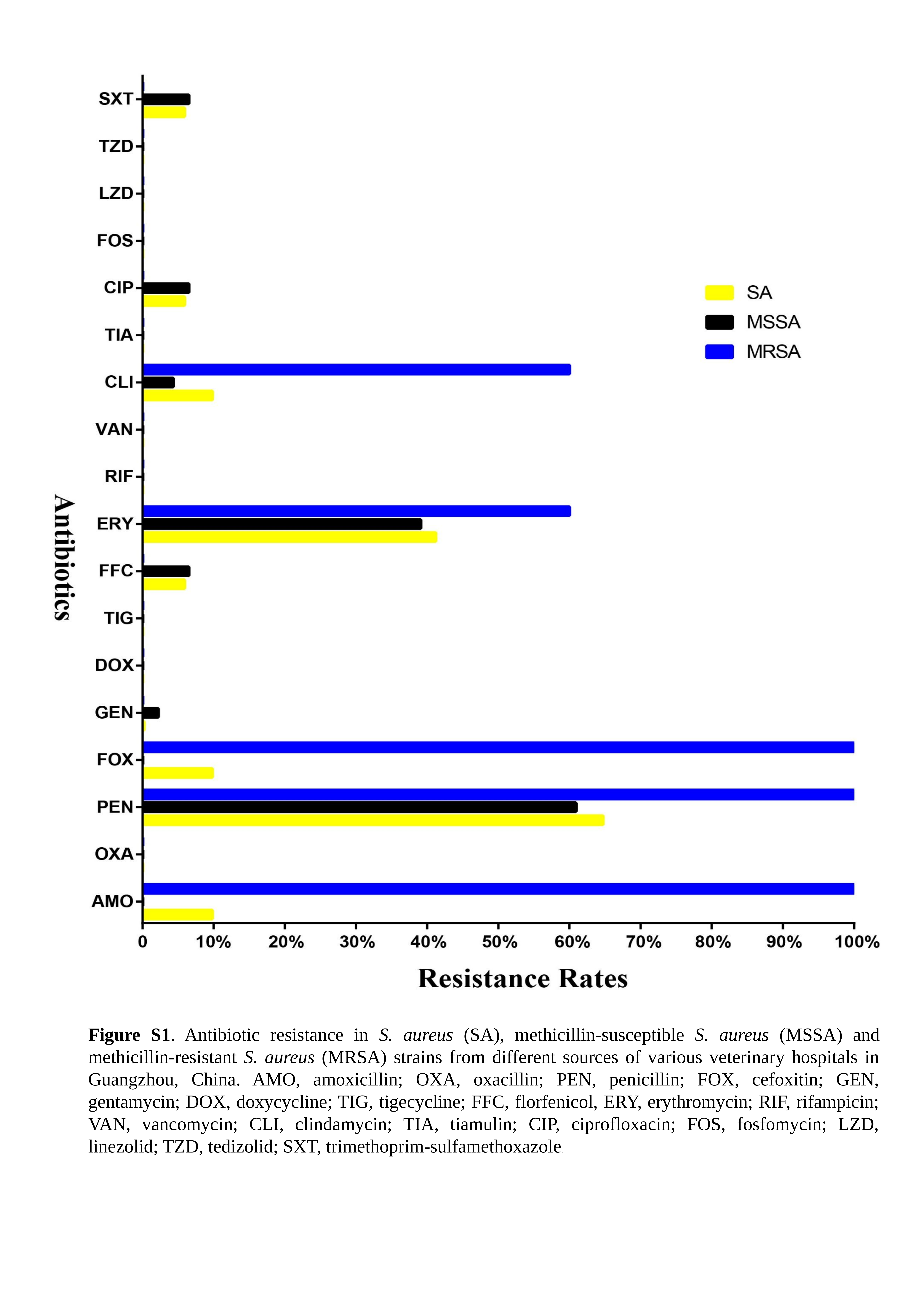

Figure S1. Antibiotic resistance in S. aureus (SA), methicillin-susceptible S. aureus (MSSA) and methicillin-resistant S. aureus (MRSA) strains from different sources of various veterinary hospitals in Guangzhou, China. AMO, amoxicillin; OXA, oxacillin; PEN, penicillin; FOX, cefoxitin; GEN, gentamycin; DOX, doxycycline; TIG, tigecycline; FFC, florfenicol, ERY, erythromycin; RIF, rifampicin; VAN, vancomycin; CLI, clindamycin; TIA, tiamulin; CIP, ciprofloxacin; FOS, fosfomycin; LZD, linezolid; TZD, tedizolid; SXT, trimethoprim-sulfamethoxazole.
