# Supplementary material for: Biofilm Production Ability, Virulence and Antimicrobial Resistance Genes in Staphylococcus aureus from Various Veterinary Hospitals
Source: Pathogens. 2020 Apr 4;9(4):264. doi: 10.3390/pathogens9040264 (PMC7238219; doi:10.3390/pathogens9040264)
Supplement: Supplementary file 1 [file pathogens-09-00264-s001.zip › Table S1.pdf]

Table S1. Primer sequences used to amplify specific virulence, antimicrobial resistance genes in this study.

Target	Gene	Primer Sequence	Tm (°C)	Amplicon Size	Positive Control Strain	Reference
Methicillin	*mecA*	F-GTGAAGATATACCAAGTGATT<br>R-ATGCGCTATAGATTGAAAGGAT	55	533	ATCC 43300[a]	[1]
	mecC	F-GAAAAAAAGGCTTAGAACGCCTC<br>R-GAAGATCTTTTCCGTTTTCAGC	54	138	MRSA 123A[a]	[2]
Oxazolidinone	*cfr*	F-TGAAGTATAAAGCAGGTTGGGAGTCA<br>R-ACCATATAATTGACCACAAGCAGC	48	746	*E. faecalis* 147C[a]	[3]
	optrA	F-AGGTGGTCAGCGAACTAA<br>R-ATCAACTGTTCCCATTCA	55	1395	*E. faecalis* 147C[a]	[4]
Vancomycin	*vanA*	F-GGCAAGTCAGGTGAAGATG<br>R-ATCAAGCGGTCAATCAGTTC	55	713	*E. faecium* A4[a]	[5]
	vanB	F-GTGACAAACCGGAGGCGAGGA<br>R-CCGCCATCCTCCTGCAAAAAA	50	430	*E. faecium* 56C[a]	[5]
Erythromycin	*ermA*	F-GTTCAAGAACAATCAATACAGAG<br>R-GGATCAGGAAAAGGACATTTTAC	47	421	MSSA 195A[a]	[6]
	ermB	F-CCGTTTACGAAATTGGAACAGGTAAAGGGC<br>R-GAATCGAGACTTGAGTGTGC	47	359	MSSA 96A[a]	[6]
	ermC	F-GCTAATATTGTTTAAATCGTCAATTCC<br>R-GGATCAGGAAAAGGACATTTTAC	47	572	MSSA 96A[a]	[6]
Tetracycline	*tetK*	F-TCGATAGGAACAGCAGTA<br>R-CAGCAGATCCTACTCCTT	57	169	MRSA 109A[a]	[2]
Aminoglycoside	*aac(6')-aph(2")*	F-TAATCCAAGAGCAATAAGGGC<br>R-GCCACACTATCATAACCACTA	55	348	MRSA 3A[a]	[7]
Leukocidin	*lukS/F-PV*	F-ATCATTAGGTAAAATGTCTGGACATGATCCA<br>R-GCATCAAGTGTATTGGATAGCAAAAGC	56	443	ATCC 49775[a]	[2]
	lukD	F-TTGCACTGCTTTTGCTATCG<br>R-GCATTTGATGTGTTGGCAAG	53	675	ATCC 33591[a]	[8]
	lukE	F-GATTGCGCCTTTAGCATCTC<br>R-GCTGAACCTGTTGGACCATT	55	612	ATCC 33591[a]	[8]
Alpha-hemolysin	*hla*	F-TGCCGCAGATTCTGATATTAA<br>R-TTTCTGAAGAACGATCTGTCCA	51	845	MRSA 13A[a]	[9]
Beta-hemolysin	*hlb*	F-TATCCAAACTGGGGGCAATA<br>R-AACCGCTTTTGAAAACATGC	50	292	NCTC 7428[b]	[8]
Delta-hemolysin	*hld*	F-TAATTAAGGAAGGAGTGATTTCAATG<br>R-TTTTTAGTGAATTTGTTCACTGTGTC	51	100	NCTC 9393[b]	[8]
Toxic shock syndrome toxin-1	*tst*	F-TTTTTTATCGTAAGCCCTTTGTTGC<br>R-CACCCGTTTTATCGCTTGAA	51	550	ATCC 33586[a]	[9]
Exfoliative toxin	*eta*	F-CTAGTGCATTTGTTATTCAAGACG<br>R-TGCATTGACACCATAGTACTTATTC	53	119	MRSA 273A[a]	[8]
	etb	F-ACGGCTATATACATTCAATTCATTG<br>R-AAAGTTATTCATTTAATGCACTGTCTC	53	262	MRSA 277A[a]	[8]
Enterotoxins	*sea*	F-CGATCAATTTATGGCTAGACGG<br>R-TTGCTTGAAGATCCAACTCCTG	50	101	ATCC 13566[b]	[10]
	seb	F-AAGGACACTAAGTTAGGGAATT<br>R-GTTACACCACCATACATACAAG	50	197	ATCC 13566[b]	[10]
	sec	F-GGTAAAGTTACAGGTGGCAAAA<br>R-TCTTGAGCTGTTGCACTTTTCT	50	170	ATCC 19095[b]	[10]
	sed	F-GTTTGATTCTTCTGATGGGTC<br>R-TGTCAATATGAAGGTGCTCTG	50	128	ATCC 23235[b]	[10]
	see	F-TAATAACCGATTGACCGAAGA<br>R-ATAACTTACCGTGGACCCTTC	50	244	ATCC 27664[b]	[10]
	seg	F-AAGTAGACATTTTTGGCGTTCC<br>R-AGAACCATCAAACTCGTATAGC	50	287	MRSA 3A[a]	[10]
	seh	F-GTCTATATGGAGGTACAACACT<br>R-GACCTTTACTTATTTCGCTGTC	53	213	ATCC 51811[b]	[10]
	sei	F-GGTGATATTGGTGTAGGTAAC<br>R-ATCCATATTCTTTGCCTTTACCAG	55	454	MRSA 3A[a]	[10]
	sej	F-ATAGCATCAGAACTGTTGTTCCG<br>R-CTTTCTGAATTTTACCACCAAAGG	55	152	MRSA46A[a]	[10]
	sek	F-TAGGTGTCTCTAATAATGCCA<br>R-TAGATATTCGTTAGTAGCTG	55	293	MRSA 238A[a]	[10]
	sel	F-TAACGGCGATGTAGGTCCAGG<br>R-CATCTATTTCTTGTGCGGTAAC	52	383	MRSA 238A[a]	[10]
	sem	F-GGATAATTCGACAGTAACAG<br>R-TCCTGCATTAAATCCAGAAC	50	379	MRSA 3A[a]	[10]
	sen	F-TATGTTAATGCTGAAGTAGAC<br>R-ATTTCCAAAATACAGTCCATA	51	282	MRSA 3A[a]	[10]
	seo	F-TGTGTAAGAAGTCAAGTGTAG	53	214	MRSA 3A[a]	[10]

		R-TCTTTAGAAATCGCTGATGA				
	icaA	F-ACACTTGCTGGCGCAGTCAA<br>R-TCTGGAACCAACATCCAACA	51	188	MSSA 96A[a]	[11]
	icaD	F-ATGGTCAAGCCCAGACAGAG<br>R-AGTATTTTCAATGTTTAAAGCAA	55	198	MSSA 96A[a]	[11]
ica operon	*icaB*	F-AGAATCGTGAAGTATAGAAAATT<br>R-TCTAATCTTTTTCATGGAATCCGT	54	880	MSSA 96A[a]	[11]
	icaC	F-ATGGGACGGATTCCATGAAAAAGA<br>R-TAATAAGCATTAATGTTCAATT	50	1066	MSSA 96A[a]	[11]
	icaR	F-ATCTAATACGCCTGAGGA<br>R-TTCTTCCACTGCTCCAA	50	205	MSSA 96A[a]	[11]
	clfA	F-ATTGGCGTGGCTTCAGTGCT<br>R-CGTTTCTTCCGTAGTTGCATTTG	53	288	ATCC 25923[a]	[11]
	clfB	F-CACTTACTTTACCGCTACTTTC<br>R-AACGAGCAATACCACTACAACAG	53	968	ATCC 25923[a]	[11]
	fnbA	F-ACCGTCAAACGCAACACAAG<br>R-TTCTGATGCCGTTCTTGGCT	53	259	ATCC 33591[a]	[11]
	fnbB	F-GTAACAGCTAATGGTCGAATTGATACT<br>R-CAAGTTCGATAGGAGTACTATGTTC	56	523	ATCC 33591[a]	[11]
	cidA	F-AGCGTAATTTCGGAAGCAACATCCA<br>R-CCCTTAGCCGGCAGTATTGTTGGTC	54	170	ATCC 33591[a]	[11]
	fib	F-CTACAACTACAATTGCGTCAACAG<br>R-GCTCTTGTAAGACCATTTTCTTCAC	54	405	ATCC 33591[a]	[11]
Adhesion	*bap*	F-CCATATATCGAAGGTGTAGAATTG<br>R-GCTGTTGAAGTTAATACTGTACCTGC	54	971	MRSA 158A[a]	[11]
	cna	F-AAAGCGTTGCCTAGTGGAGA<br>R-AGTGCCTTCCCAAACCTTTT	50	192	ATCC 33591[a]	[11]
	ebps	F-AGAATGCTTTTGCAATGGAT<br>R-AATATCGCTAATGCACCGAT	51	652	ATCC 25923[a]	[11]
	eno	F-TGCCGTAGGTGACGAAGGTGGTT<br>R-GCACCGTGTTCGCCTTCGAACT	50	196	ATCC 33591[a]	[11]
	sdrC	F-ACGACTATTAAACCAAGAAC<br>R-GTACTTGAAATAAGCGGTTG	50	560	ATCC 33591[a]	[11]
	sdrD	F-GGAATAAAGTTGAAGTTTC<br>R-ACTTTGTCATCAACTGTAAT	52	500	ATCC 33591[a]	[11]
	sdrE	F-CAGTAAATGTGTCAAAAGA<br>R-TTGACTACCAGCTATATC	52	767	ATCC 33591[a]	[11]
Others	*nuc*	F-GCGATTGATGGTGATACGGTT<br>R-AGCCAAGCCTTGACGAACTAAAGC	55	270	ATCC 29213[a]	[2]
	spa	F-TAAAGACGATCCTTCGGTGAGC<br>R-CAGCAGTAGTGCCGTTTGCTT	60	-	ATCC 29213[a]	[6]

ATCC: American Type Culture Collection; MRSA: methicillin-resistant *Staphylococcus aureus*; *E. faecalis*: *Enterococcus faecalis*; *E. faecium*: *Enterococcus faecium*; MSSA: methicillin-susceptible *Staphylococcus aureus*; NCTC: National Collection of Type Cultures.

[a] These strains were obtained from the college of veterinary medicine of south China agricultural university. Except for ATCC strains, all strains were completely sequenced using Illumina Hiseq TM2000 sequencing platform.

[b] These strains were obtained from the China General Microbiological Culture Collection Center.

References

1. Zhang, K.Y.; McClure, J.A.; Elsayed, S.; Louie, T.; Conly, J.M. Novel multiplex PCR assay for characterization and concomitant subtyping of staphylococcal cassette chromosome mec types I to V in methicillin-resistant *Staphylococcus aureus*. *J Clin Microbiol*. **2005**, *43*, 5026–5033.
2. Dweba, C.C.; Zishiri, O.T.; El Zowalaty, M.E. Isolation and Molecular Identification of Virulence, Antimicrobial and Heavy Metal Resistance Genes in Livestock-Associated Methicillin-Resistant *Staphylococcus aureus*. *Pathogens*. **2019**, *8*.
3. Kehrenberg, C.; Schwarz, S. Distribution of florfenicol resistance genes *fexA* and *cfr* among chloramphenicol-resistant *Staphylococcus* isolates. *Antimicrob Agents Chemother*. **2006**, *50*, 1156–1163.
4. Wang, Y.; Lv, Y.A.; Cai, J.C.; Schwarz, S.; Cui, L.Q.; Hu, Z.D.; Zhang, R.; Li, J.; Zhao, Q.; He, T.; et al. A novel gene, *optrA*, that confers transferable resistance to oxazolidinones and phenicols and its presence in *Enterococcus faecalis* and *Enterococcus faecium* of human and animal origin. *J Antimicrob Chemother*. **2015**, *70*, 2182–2190.
5. Al-Amery, K.; Elhariri, M.; Elsayed, A.; El-Moghazy, G.; Elhelw, R.; El-Mahallawy, H.; El Hariri, M.; Hamza, D. Vancomycin-resistant *Staphylococcus aureus* isolated from camel meat and slaughterhouse workers in Egypt. *Antimicrob Resist Infect Control*. **2019**, *8*, 129.
6. Li, X.; Huang, T.; Xu, K.; Li, C.; Li, Y. Molecular characteristics and virulence gene profiles of *Staphylococcus aureus* isolates in Hainan, China. *BMC Infect Dis*. **2019**, *19*, 873.
7. Khosravi, A.D.; Jenabi, A.; Montazeri, E.A. Distribution of genes encoding resistance to aminoglycoside modifying enzymes in methicillin-resistant S*taphylococcus aureus* (MRSA) strains. *Kaohsiung J Med Sci*. **2017**, *33*, 587–593.
8. Shukla, S.K.; Karow, M.E.; Brady, J.M.; Stemper, M.E.; Kislow, J.; Moore, N.; Wroblewski, K.; Chyou, P.H.; Warshauer, D.M.; Reed, K.D.; et al. Virulence genes and genotypic associations in nasal carriage, community-associated methicillin-susceptible and methicillin-resistant USA400 *Staphylococcus aureus* isolates. *J Clin Microbiol*. **2010**, *48*, 3582–3592.
9. Ote, I.; Taminiau, B.; Duprez, J.N.; Dizier, I.; Mainil, J.G. Genotypic characterization by polymerase chain reaction of *Staphylococcus aureus* isolates associated with bovine mastitis. *Vet Microbiol*. **2011**, *153*, 285–292.
10. Wu, Y.H.; Li, J.; Qiao, M.F.; Meng, D.; Meng, Q.L.; Qiao, J.; Zhang, X.X.; Wang, L.X.; Cai, K.J.; Zhang, J.S.; et al. Characteristic profiles of biofilm, enterotoxins and virulence of *Staphylococcus aureus* isolates from dairy cows in Xinjiang Province, China. *J Vet Sci*. **2019**, *20*, e74
11. Chen, Q.; Xie, S.M.; Lou, L.Q.; Cheng, S.; Liu, X.D.; Zheng, W.; Zheng, Z.B.; Wang, H.Q. Biofilm formation and prevalence of adhesion genes among *Staphylococcus aureus* isolates from different food sources. *Microbiologyopen*. **2020**, *9*, e00946.
